# Embryo-secreted microRNAs enable non-invasive assessment of chromosomal status and viability in *in vitro* fertilization

**DOI:** 10.3389/fcell.2025.1747211

**Published:** 2026-01-05

**Authors:** Masoumeh Esmaeilivand, Mohammad Hossein Nasr-Esfahani

**Affiliations:** 1 Department of Obstetrics and Gynecology, School of Medicine, Kermanshah University of Medical Sciences, Kermanshah, Iran; 2 Department of Animal Biotechnology, Reproductive Biomedicine Research Center, Royan Institute for Biotechnology, ACECR, Isfahan, Iran

**Keywords:** aneuploidy, embryo selection, implantation, IVF, microRNA, non-invasive biomarkers

## Abstract

Selecting embryos with the highest developmental potential remains a decisive step for achieving successful *in vitro* fertilization outcomes. Conventional evaluation methods such as morphological grading, time-lapse imaging, and preimplantation genetic testing for aneuploidy provide valuable information but are limited by subjectivity, invasiveness, and cost. These challenges have driven increasing interest in non-invasive biomarkers capable of improving the precision and safety of embryo assessment. Emerging evidence indicates that human embryos actively release microRNAs into their surrounding environment, particularly into the spent culture medium and blastocoel fluid. These extracellular microRNAs regulate pathways involved in cell cycle progression, apoptosis, differentiation, and embryo-endometrium communication. Distinct expression signatures, including miR-21-5p, miR-661, and members of the miR-17∼92 cluster, correlate with chromosomal integrity and implantation competence, suggesting their potential as complementary or alternative biomarkers to trophectoderm biopsy. Comparative analyses across digital PCR, quantitative RT-PCR, microarray, and next-generation sequencing platforms reveal both methodological advances and unresolved challenges, such as low RNA yield, contamination risk, and inconsistent normalization strategies. Embryo-derived microRNAs thus represent a promising avenue for non-invasive embryo evaluation, with future clinical translation hinging on robust multicenter validation and integration with imaging, metabolomics, and artificial intelligence–based analytics.

## 1 Introduction

In assisted reproductive technology (ART), embryo selection remains one of the most crucial determinants of IVF success, directly influencing implantation and live birth rates. Traditional methods, such as morphological grading and time-lapse imaging, provide useful morphological insights but have limited predictive accuracy and cannot reliably distinguish euploid from aneuploid embryos (Wang et al., 2023; Zou et al., 2024; Sciorio et al., 2025). Preimplantation genetic testing for aneuploidy (PGT-A) offers direct chromosomal assessment and has improved selection efficiency; however, it is invasive, costly, and occasionally discordant with the embryo’s true genetic state due to mosaicism (Navarro-Sánchez et al., 2022; Cinnioglu et al., 2023; Huang et al., 2023). These limitations have prompted the search for non-invasive biomarkers that can complement or even replace invasive testing. Among the candidates, microRNAs (miRNAs) have recently attracted considerable attention. MiRNAs are short, non-coding RNA molecules (∼22 nucleotides) that regulate gene expression post-transcriptionally by targeting messenger RNAs, thereby controlling essential cellular processes such as proliferation, apoptosis, and differentiation (Esmaeilivand et al., 2024). Importantly, preimplantation embryos secrete miRNAs into their surrounding microenvironment, including spent culture medium (SCM) and blastocoel fluid (BF), making these molecules accessible for non-invasive analysis (Esmaeilivand et al., 2022b).

Several studies have reported variable expression of embryo-derived miRNAs associated with chromosomal status and implantation potential. For example, certain miRNA signatures detected in spent culture medium (SCM) have been correlated with blastocyst viability and developmental competence (Esmaeilivand et al., 2022a; Omes et al., 2024; Caponnetto et al., 2025) while profiling of BF has revealed distinct expression patterns related to chromosomal abnormalities (Esmaeilivand et al., 2022b). Despite these promising findings, significant challenges remain, including low RNA yield, contamination from maternal cells or culture reagents, and the absence of standardized analytical protocols (Jusic et al., 2024; Mukerjee et al., 2025). Therefore, a comprehensive synthesis of current evidence is warranted to clarify the clinical potential of embryo-secreted miRNAs. Relevant literature was identified through searches in PubMed, Scopus, and Web of Science (supplemented by manual searches in Google Scholar). The search covered January 2014 to September 2025 and included studies focusing on embryo-derived microRNAs in spent culture medium, blastocoel fluid, or trophectoderm biopsy associated with aneuploidy, embryo viability, or implantation outcomes. After screening and eligibility assessment, 42 studies were included in this review.

This review aims to summarize the role of embryo-secreted miRNAs as non-invasive biomarkers of embryo viability and chromosomal integrity, highlight recent methodological advances, and outline future directions toward their integration into routine IVF practice.

## 2 Sources of embryo-secreted microRNAs

**FIGURE 1:**
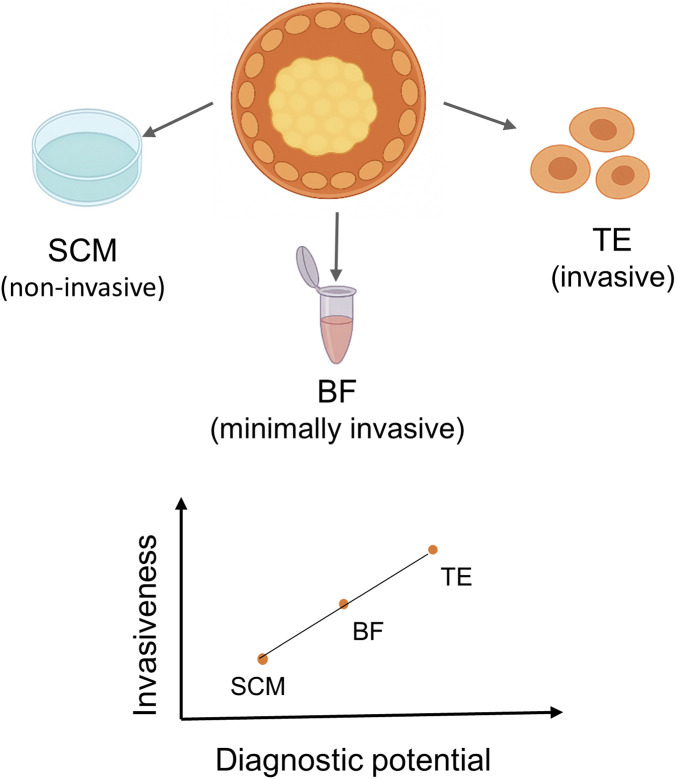
Sources of embryo-derived microRNAs in IVF. Comparative schematic of biological sources of embryo-derived microRNAs (miRNAs) used in IVF research. Spent culture medium (SCM) and blastocoel fluid (BF) represent non-invasive or minimally invasive sampling methods that directly reflect embryo secretions. Trophectoderm (TE) biopsy, follicular fluid, and endometrial or uterine fluid are additional materials; however, these are either invasive or indirectly related to embryo physiology.

MicroRNAs (miRNAs) from the embryo have been discovered to be secreted from a variety of biological compartments associated with *in vitro* fertilization (IVF), providing a range of non-invasive research opportunities (Esmaeilivand et al., 2022a). Spent culture medium (SCM) and blastocoel fluid (BF), which are direct secretions from the developing embryo, are two sources that have been widely explored (Esmaeilivand et al., 2022b; Campos and Nel-Themaat, 2024; Caponnetto et al., 2025). Additional sources, such as trophectoderm (TE) biopsy samples, follicular fluid, and endometrial or uterine fluid, have also been explored, though these are either invasive or indirectly reflect embryo physiology (Ibañez-Perez et al., 2022; Aoki et al., 2024; Esmaeilivand et al., 2024). The relative invasiveness and diagnostic value of these biological sources are illustrated in Figure 1.

### 2.1 Spent culture medium (SCM)

Embryos, as living organisms, are capable of secreting and releasing a variety of biological molecules, including microRNAs (miRNAs), into their surrounding culture medium. Several studies have demonstrated that the miRNA profile in spent culture medium (SCM) is linked to blastocyst morphology, developmental competence, and implantation potential. Therefore, determining the miRNA pattern in spent culture medium (SCM) can be used as a non-invasive biomarker to assess embryo quality *in vitro* fertilization (IVF) processes (Abu-Halima et al., 2020; Ovčar and Kovačič, 2024). However, the amount of RNA released into the culture medium is very low, which makes its extraction and accurate identification challenging. On the other hand, the possibility of sample contamination with RNA from peripheral cells, such as cumulus cells surrounding the oocyte or embryo, as well as compounds present in laboratory reagents, can lead to inaccurate or ambiguous results. Therefore, the precise isolation of embryo-specific miRNAs from other sources must be carefully performed to ensure reliable analyses (Voros et al., 2025c). Different biological sources of embryo-secreted miRNAs, together with their main advantages, limitations, and clinical relevance, are summarized in Table 1.

**TABLE 1:** Sources of embryo-derived microRNAs: key advantages, limitations, and level of clinical evidence.

Source	Advantages	Limitations	Clinical evidence/Reported associations	Type of evidence/Study design
Spent culture medium (SCM)	Direct secretion from embryos; non-invasive; easy to collect during IVF culture	Low RNA yield; risk of contamination from maternal cells or culture reagents	Associations with blastocyst morphology, embryo viability, implantation and pregnancy outcomes reported across multiple studies	Observational cohort studies; targeted miRNA profiling (qRT-PCR/microarray)
Blastocoel fluid (BF)	Enriched in extracellular vesicles and miRNAs; closely reflects embryo physiology	Extremely small volume; technically challenging extraction and analysis	Distinct miRNA profiles reported between euploid and aneuploid blastocysts; associations with implantation potential	Observational comparative studies (euploid vs. aneuploid embryos)
Trophectoderm (TE) biopsy	Provides molecular validation; gold standard for chromosomal assessment	Invasive; not suitable as a routine non-invasive biomarker	Widely used for PGT-A; confirms molecular patterns observed in SCM and/or BF	Clinical diagnostic studies (PGT-A-based analyses)
Follicular fluid	Reflects oocyte microenvironment; miRNAs involved in oocyte maturation and fertility	Mixed maternal and oocyte origin; limited embryo specificity	Altered miRNA profiles associated with PCOS and infertility-related conditions	Observational studies in infertility populations
Endometrial/uterine fluid	Non-invasive sampling; provides insight into implantation environment	Mixture of maternal and embryonic signals; limited specificity for embryo origin	miRNA profiles linked to endometrial receptivity and implantation success	Exploratory observational studies; receptivity-focused analyses

For SCM, and BF, current evidence is largely observational and exploratory, highlighting the need for standardized protocols and prospective clinical validation before routine clinical application.

### 2.2 Blastocoel fluid (BF)

During blastocyst development, the blastocoel cavity enlarges, and the fluid within it contains extracellular vesicles enriched with miRNAs. These vesicles play a crucial role in cellular signaling and gene expression regulation during early embryonic development, indicating the presence of complex regulatory mechanisms that are initiated by the embryo (Rule et al., 2018; Esmaeilivand et al., 2022b; Campos and Nel-Themaat, 2024). Studies have shown that miRNA profiles in BF can show significant differences between euploid and aneuploid embryos. These differences imply the potential of BF to provide reliable and non-invasive biomarkers for the detection of chromosomal status and embryo competence (Battaglia et al., 2019; Esmaeilivand et al., 2022b). While the extraction and analysis of miRNA and DNA from blastocoel fluid (BF) is challenging due to the small sample size and the need for sensitive and precise methods, with the advancement of molecular technologies, this pathway has been proposed as a new strategy in assisted reproductive technology (ART) to improve the selection of high-quality embryos and increase the implantation success rate (Frisendahl et al., 2025).

### 2.3 Trophectoderm (TE) biopsy

Trophectoderm (TE) biopsy is used for preimplantation genetic testing (PGT-A). Although it is an invasive procedure, it remains the gold standard for genetic health screening and detection of chromosomal abnormalities in IVF (Tomic et al., 2022; Voros et al., 2025a). Some studies have shown that microRNA and mRNA can be extracted from trophectoderm samples, and specific molecular patterns associated with aneuploidy and implantation success can be identified (Esmaeilivand et al., 2024). This provides important biological evidence in confirming embryo-derived microRNAs detected in non-invasive samples such as blastocoel fluid (BF) or culture medium. This means that molecular findings from TE biopsy provide biological validity to data obtained from less invasive samples (Voros et al., 2025a). However, TE biopsy itself, due to its invasive nature, cannot replace non-invasive methods in clinical practice (Tomic et al., 2022).

### 2.4 Other sources

Follicular fluid is the environment in which the oocyte grows and develops and contains a collection of microRNAs. These miRNAs regulate gene expression in various pathways that control oocyte maturation, health, and fertility (Feng et al., 2015; Aoki et al., 2024). In diseases such as polycystic ovary syndrome, the microRNA profile in follicular fluid is altered, which can lead to reduced oocyte quality (Butler et al., 2019). In addition, the cumulus cells that surround the oocyte also produce and secrete microRNAs and extracellular vesicles containing these RNAs, which act as molecular messengers for communication with the oocyte and help regulate signaling pathways related to oocyte growth, maturation, and fertilization (Da Broi et al., 2018). Endometrial fluid is another important source of microRNAs that provide the uterine environment for embryo implantation. miRNAs in this fluid may play a role in the regulatory processes of implantation and embryo maintenance in the uterus; changes in the expression of these miRNAs may affect implantation success and pregnancy health (Voros et al., 2025c). While these fluids provide valuable insights into the reproductive microenvironment, they represent a mixture of maternal and embryonic contributions, which limits their specificity as embryo-derived biomarkers (Da Broi et al., 2018; Voros et al., 2025c). Nevertheless, these non-embryo-specific sources may hold promise in multi-omics strategies that integrate both embryonic and maternal signals for a more comprehensive assessment of implantation potential (Voros et al., 2025c).

## 3 miRNAs and chromosomal aneuploidy

Recent research has highlighted embryo-secreted microRNAs as promising indicators of chromosomal status (Almutlaq et al., 2024; Caponnetto et al., 2025; Toporcerová et al., 2025). Rosenbluth et al. (2014) reported that miR-191 levels were increased in the culture medium of aneuploid embryos. This miRNA has been associated with activation of the PI3K/AKT and MAPK pathways in other cellular models, suggesting a possible role in promoting cell survival and reducing apoptosis, which may allow chromosomally abnormal embryos to persist rather than undergo natural elimination. (Rosenbluth et al., 2014). Esmaeilivand et al. (2022a) reported differential expression of miR-661 and miR-20a between euploid and aneuploid blastocysts in blastocoel fluid (BF) (Esmaeilivand et al., 2022b). In addition, Omes et al. (2024) reported specific miRNAs, such as miR-21-5p and miR-661, in spent culture media that were linked not only to embryo developmental potential but also suggested a possible link to chromosomal constitution (Omes et al., 2024). Studies suggest that miR-20a plays an important role in G1/S progression by modulating the E2F/Cyclin D1 axis; its increased expression may lead to accelerated and insufficient passage through cell-cycle checkpoints (Trompeter et al., 2011). miR-661 has been linked to reduced cell adhesion and induction of epithelial-mesenchymal transition (EMT) in several cellular models, reflecting cellular instability and stress responses that may parallel chromosomal imbalance in embryos (Cuman et al., 2015). In addition, miR-21-5p may influence apoptosis-related signaling by targeting PTEN and modulating the PI3K/AKT pathway, as reported in other cellular contexts, which could support cell survival in embryos with chromosomal abnormalities (Zhang et al., 2015).

**FIGURE 2:**
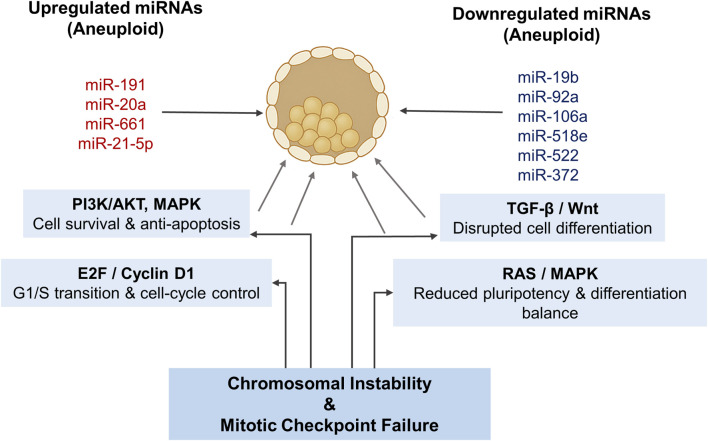
Embryo-secreted microRNAs associated with chromosomal aneuploidy and checkpoint dysregulation. This schematic summarizes the dysregulation of embryo-secreted microRNAs (miRNAs) in aneuploid embryos and their involvement in stress-related and developmental signaling pathways. Upregulated miRNAs (miR-191, miR-20a, miR-661, and miR-21-5p) are linked to the activation of the PI3K/AKT–MAPK and E2F/Cyclin D1 axes, promoting cell survival, suppressing apoptosis, and accelerating G1/S transition through incomplete checkpoint control. miR-661 also contributes to epithelial instability and induction of EMT. Conversely, downregulated miRNAs (miR-19b, miR-92a, miR-106a, miR-518e, miR-522, and miR-372) normally regulate Cyclins/CDKs, TGF-β/Wnt, and RAS/MAPK signaling cascades that maintain pluripotency and balanced differentiation; their suppression may disrupt these processes, leading to asynchrony, abnormal differentiation, and chromosomal segregation errors. Collectively, these alterations converge on aneuploidy maintenance and checkpoint failure, favoring the persistence of chromosomally abnormal embryos despite intrinsic surveillance mechanisms.

Also, Almutlaq et al. (2024) in their systematic review, reported that miR-19b, miR-517c, miR-518e, miR-522, miR-92a, and miR-106a were consistently downregulated in aneuploid blastocysts, reinforcing the significant association between miRNA patterns and chromosomal errors (Aoki et al., 2024). Among these, miR-19b, miR-92a, and miR-106a are members of the miR-17∼92 family, which has been suggested to regulate cell-cycle progression by modulating Cyclins and CDKs, as well as TGF-β and Wnt signaling pathways. Reduced expression of these miRNAs may impair checkpoint control and contribute to irregular chromosomal segregation (Concepcion et al., 2012). Esmaeilivand et al. (2024) analyzed trophectoderm biopsies and showed that miR-30c and miR-372 were downregulated in aneuploid blastocysts. miR-30c has been suggested to modulate the Wnt/β-catenin pathway involved in blastocyst development and differentiation, and its reduced expression may disrupt the coordination of cell division. On the other hand, miR-372, which belongs to the RAS/MAPK and TGF-β signaling axes, has been suggested to participate in maintaining the pluripotency of embryonic cells. Its reduced expression in aneuploid embryos may indicate a loss of the natural balance between pluripotency and differentiation (Esmaeilivand et al., 2024). The embryo-secreted microRNAs associated with chromosomal aneuploidy and checkpoint dysregulation are shown in Figure 2. Key miRNAs implicated in aneuploidy, their signaling pathways, and reported biological effects are listed in Table 2.

**TABLE 2:** Key embryo-derived miRNAs associated with aneuploidy and their biological pathways.

miRNA	Primary pathway/Target	Functional role/Biological effect	Supporting references
miR-191	PI3K/AKT, MAPK	Promotes cell survival, inhibits apoptosis → persistence of abnormal embryos	Rosenbluth et al., 2014; Cuman et al., 2015
miR-661	EMT, cell adhesion	Reduces cell adhesion and promotes instability; marker of cellular stress and aneuploidy	Esmaeilivand et al., 2022b; Cuman et al., 2015; Omes et al., 2024
miR-21-5p	PTEN/PI3K/AKT	Anti-apoptotic; enhances survival of chromosomally abnormal embryos	Omes et al., 2024; Zhang et al., 2015
miR-20a	E2F/Cyclin D1 axis	Dysregulated G1/S transition → abnormal cell division and chromosomal missegregation	Esmaeilivand et al., 2022b; Trompeter et al., 2011
miR-19b, miR-92a, miR-106a *(miR-17∼92 cluster)*	Cyclins/CDKs, TGF-β, Wnt	Regulate cell-cycle checkpoints; downregulation causes segregation errors in aneuploidy	Almutlaq et al., 2024; Concepcion et al., 2012
miR-30c	Wnt/β-catenin	Controls blastocyst differentiation; reduced expression disrupts cell-division coordination	Esmaeilivand et al. (2024)
miR-372	RAS/MAPK, TGF-β	Maintains pluripotency; downregulation leads to imbalance between pluripotency and differentiation	Esmaeilivand et al. (2024)

## 4 miRNAs and implantation potential

Successful implantation depends on a complex interplay between embryo competence and endometrial receptivity. Even chromosomally normal embryos may fail to implant due to inadequate molecular signaling or impaired embryo–endometrium crosstalk (Kim and Kim, 2017; Lacconi et al., 2024). Recent studies suggest that microRNAs (miRNAs) secreted by embryos and present in maternal reproductive fluids may serve as biomarkers of implantation potential (Mutia et al., 2023; Qi et al., 2023).

Rosenbluth et al. (2014) demonstrated that human embryos release miR-191 into the culture medium and that its levels are associated with implantation outcomes. This miRNA has been linked to activation of the PI3K/AKT and MAPK pathways, which promote cell survival and inhibit apoptosis, potentially supporting the persistence of embryos capable of implantation (Rosenbluth et al., 2014). In addition, Battaglia et al. (2019) showed that miRNAs in blastocoel fluid (BF) are connected to the Wnt/β-catenin and RAS/MAPK pathways and that changes in their expression can disrupt the balance between pluripotency and differentiation and affect implantation capacity (Battaglia et al., 2019).

Berkhout et al. (2020) reported that high-quality embryos secrete miR-320a, this miRNA enhances the migration of endometrial stromal cells (hESCs) and prepares the receptive field for implantation through cytoskeletal rearrangement and activation of genes related to cell motility (Berkhout et al., 2020). Wang et al. (2021) identified three miRNAs—miR-199a-5p, miR-483-5p, and miR-432-5p—in the spent culture medium (SCM) of human embryos, whose expression was associated with successful implantation. Pathway analysis suggested that these miRNAs were involved in cell-cycle regulation and apoptosis, indicating their potential as markers of blastocyst growth and viability (Wang et al., 2021). Kamijo et al. (2022) analyzed more than 60 blastocysts and detected 53 distinct miRNAs. Eight miRNAs, including miR-191-5p, miR-320a, miR-92a-3p, miR-509-3p, miR-378a-3p, miR-28-3p, miR-512-5p, and miR-181a-5p, showed the strongest association with implantation outcomes. Pathway enrichment analysis indicated that these miRNAs are involved in PI3K/AKT, MAPK/ERK, cell-cycle, and survival pathways, suggesting that their coordinated activity may influence implantation potential (Kamijo et al., 2022). Recently, Caponnetto et al. (2025) compared miRNAs derived from blastocoel fluid (BF) and spent culture medium (SCM), revealing distinct expression patterns between the two sources. Some miRNAs specific to spent culture medium (SCM) were associated with endometrial receptivity, adhesion, and invasion pathways (Caponnetto et al., 2025).

**FIGURE 3:**
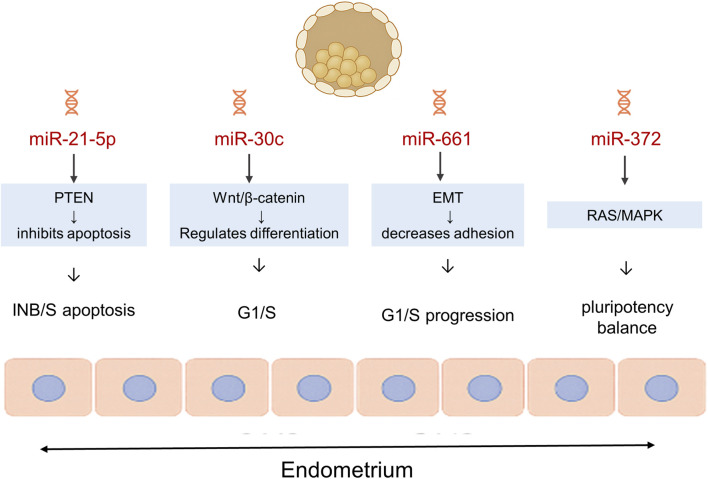
Schematic representation of miRNA-mediated embryo–endometrium signaling during implantation. Diagram showing bidirectional molecular communication between the blastocyst and the endometrium mediated by embryo-derived microRNAs (miRNAs). miR-21-5p is suggested to regulate the PI3K/AKT pathway, thereby inhibiting apoptosis and promoting cell survival. miR-30c has been suggested to modulate the Wnt/β-catenin signaling axis involved in blastocyst differentiation. miR-661 has been suggested to modulate epithelial-mesenchymal transition (EMT) and reduce cell adhesion at the implantation interface, potentially contributing to the regulation of local stress responses. miR-20a, a member of the miR-17∼92 cluster, is thought to modulate E2F/Cyclin D1 and TGF-β/MAPK signaling pathways to coordinate G1/S-phase cell-cycle progression. miR-372 may act through the RAS/MAPK and TGF-β signaling axes to help maintain the balance between pluripotency and differentiation within the endometrial environment. Together, these miRNAs appear to orchestrate adhesion, proliferation, and differentiation pathways that support embryo–endometrium communication and implantation success.

Additionally, studies have shown that uterine fluid miRNA profiles are disrupted in women with recurrent implantation failure, suggesting a disruption in the embryo-endometrial communication (von Grothusen et al., 2022). Similarly, specific miRNA expression in endometrial fluid can non-invasively identify an implanting (receptive) endometrium (Ibañez-Perez et al., 2022). In addition, distinct plasma miRNA patterns during the preimplantation period are associated with biochemical pregnancy loss after embryo transfer, reinforcing the role of systemic signaling of miRNAs in early pregnancy outcomes (Shen et al., 2024). However, the molecular overlap between implantation-related miRNAs and those linked to general embryo stress complicates their interpretation. For example, Soczewski et al. (2023) reported that miR-21-5p (among others) is involved in both stress/inflammatory pathways and implantation-associated signaling, highlighting this overlap(Soczewski et al., 2023). A schematic representation of the main embryo and endometrium-derived miRNAs and their signaling interactions during implantation is shown in Figure 3. miRNAs associated with implantation potential, their roles, and sample origins are presented in Table 3.

**TABLE 3:** Key microRNAs associated with implantation potential.

miRNA	Source/Sample	Main pathway/Function	Role in implantation	Supporting references
miR-191	Spent culture medium (SCM)	PI3K/AKT, MAPK	Promotes embryo survival and implantation competence through anti-apoptotic signaling	Rosenbluth et al. (2014)
miR-320a	Spent culture medium (SCM)	Cytoskeletal and migration genes	Secreted by high-quality embryos; enhances migration of endometrial stromal cells and receptivity	Berkhout et al. (2020)
miR-199a-5p	SCM	Cell-cycle, apoptosis regulation	Associated with successful implantation and improved blastocyst development	Wang et al. (2021)
miR-483-5p	SCM	Apoptosis regulation	Positively correlates with embryo viability and implantation outcome	Wang et al. (2021)
miR-432-5p	SCM	Cell-cycle progression	Marker of developmental competence and implantation success	Wang et al. (2021)
miR-92a-3p	Blastocoel fluid/SCM	PI3K/AKT, MAPK	Strongly linked to implantation outcome; participates in survival and adhesion pathways	Kamijo et al. (2022)
miR-509-3p	Blastocoel fluid	PI3K/AKT	Correlated with implantation success; may regulate embryo–endometrium crosstalk	Kamijo et al. (2022)
miR-378a-3p	Blastocoel fluid	MAPK/ERK	Contributes to cellular proliferation and differentiation supporting implantation	Kamijo et al. (2022)
miR-28-3p	Blastocoel fluid	Cell-cycle control	Associated with embryo quality and implantation potential	Kamijo et al. (2022)
miR-512-5p	Blastocoel fluid	TGF-β/survival	Positively linked to implantation competence	Kamijo et al. (2022)
miR-181a-5p	Blastocoel fluid	MAPK, cell adhesion	Supports embryo–endometrial communication and trophoblast invasion	Kamijo et al. (2022)
miR-21-5p	Endometrium/plasma	PTEN/PI3K/AKT	Dual role in stress and implantation; modulates inflammatory and apoptotic responses	Soczewski et al., 2023; Shen et al., 2024
miR-21-5p, miR-661	SCM/BF	Endometrial receptivity and adhesion	Indicate implantation competence; involved in invasion and attachment processes	Omes et al., 2024; Caponnetto et al., 2025
miR-191-5p, miR-320a, miR-92a-3p	SCM/BF	PI3K/AKT, MAPK, cell-cycle	Co-regulated network supporting implantation success	Kamijo et al., 2022; Caponnetto et al., 2025
Uterine fluid miRNAs *(various)*	Uterine fluid	Receptivity pathways	Dysregulated in recurrent implantation failure	von Grothusen et al. (2022)
Endometrial fluid miRNAs *(various)*	Endometrial fluid	Implantation receptivity	Identify receptive vs. non-receptive endometrium	Ibañez-Perez et al. (2022)

## 5 Methods of miRNA profiling in IVF

Accurate detection and quantification of embryo-derived microRNAs in non-invasive samples such as spent culture medium (SCM) or blastocoel fluid (BF) remain challenging because of the extremely low RNA content and limited sample volume (Roest et al., 2021). To overcome these limitations, several analytical platforms have been developed, each offering distinct advantages and constraints. Among them, qRT-PCR remains the most widely used because of its high sensitivity, specificity, and relatively low cost. This approach enables detailed analysis of known miRNAs but has limited capacity to identify novel or low-abundance species (Kroh et al., 2010). Microarray technology allows simultaneous screening of hundreds of miRNAs and offers broader coverage than qRT-PCR, although its lower sensitivity and background hybridization noise can affect data quality (Wang et al., 2012). Next-generation sequencing (NGS) provides the most comprehensive overview, enabling unbiased discovery of known and novel miRNAs, detection of isoforms, and identification of sequence variants. However, it requires specialized bioinformatics expertise, high-quality input material, and remains expensive. Building on these approaches, digital PCR (dPCR) has recently been introduced for absolute quantification of embryo-derived miRNAs, offering higher precision than conventional qRT-PCR (Whale et al., 2012). Although not yet widely adopted in reproductive medicine, dPCR shows strong potential for rapid and clinically scalable applications (Busato et al., 2025).

Regardless of the platform used, challenges such as low RNA extraction efficiency, risk of contamination, lack of consensus on normalization, and absence of standardized laboratory protocols still hinder reproducibility and inter-study comparisons (Moldovan et al., 2014; Roest et al., 2021). Overall, developing standardized procedures for isolation, quantification, and analysis of miRNA data is a key prerequisite for integrating these technologies into routine IVF practice. The advantages, limitations, and recommended applications of the major miRNA profiling methods are summarized in Table 4.

**TABLE 4:** Comparison of methods for profiling embryo-derived microRNAs in IVF**.**

Method	Advantages	Limitations	Typical applications in IVF research	Current clinical readiness
qRT-PCR	Very high sensitivity and specificity; relatively low cost; widely available instruments; short turnaround time	Limited to known miRNAs; low throughput; depends on normalization strategy	Targeted quantification and validation of candidate miRNAs in SCM or BF	Research and targeted validation
Microarray	Simultaneous screening of hundreds of miRNAs; broader coverage than qRT-PCR	Lower sensitivity for low-abundance miRNAs; cross-hybridization; semi-quantitative	Exploratory and comparative studies (e.g., euploid vs. aneuploid embryos)	Discovery-phase only
Next-generation sequencing (NGS)	Unbiased detection of known and novel miRNAs; identification of isoforms and sequence variants; comprehensive profiling	High cost; complex library preparation; requires advanced bioinformatics expertise; longer turnaround time	Biomarker discovery; comprehensive miRNA landscape characterization	Discovery-phase only
Digital PCR (dPCR)	Absolute quantification; very high precision and sensitivity for low-abundance targets; minimal dependence on reference genes	Limited availability; higher cost per assay; not yet standardized for IVF samples	Rapid quantification of selected miRNA panels; potential same-cycle embryo assessment	Near-clinical translational potential

While qRT-PCR, remains the most commonly used platform in IVF-related miRNA, studies, emerging technologies such as dPCR, offer improved precision and faster turnaround times, making them promising candidates for future clinical implementation following multicenter validation and standardization.

## 6 Limitations and challenges

Although microRNAs have emerged as promising non-invasive biomarkers for assessing embryo viability and chromosomal status, several obstacles still prevent their entry into routine use in IVF. Embryo-derived miRNAs are present at extremely low levels, often near the detection limits of current assays (Kroh et al., 2010; Moldovan et al., 2014; Roest et al., 2021). Variability in RNA extraction efficiency, reverse transcription, and amplification introduces technical noise and compromises reproducibility (Moldovan et al., 2014). Spent culture medium (SCM) and blastocoel fluid (BF) are often contaminated by maternal cells, sperm remnants, or culture reagents, so distinguishing embryo-specific miRNAs from background signals therefore remains a critical challenge (Rosenbluth et al., 2013; Roest et al., 2021).

Studies employ different RNA isolation kits, input volumes, and normalization strategies. This heterogeneity limits comparability across studies, thereby preventing meta-analysis (Moldovan et al., 2014). Embryo-to-embryo heterogeneity and developmental stage–specific expression patterns further complicate interpretation (Esmaeilivand et al., 2022b). Moreover, some miRNAs associated with aneuploidy or implantation failure overlap with stress-induced miRNAs, undermining specificity (Soczewski et al., 2023). Most studies to date have small sample sizes and lack prospective validation (Caponnetto et al., 2025). Consequently, predictive accuracy remains moderate, and no consensus panel of miRNAs has yet been established. The integration of miRNA testing into IVF workflows would require robust, rapid, and cost-effective assays. Current technologies, such as NGS, are costly and time-consuming, thus posing barriers to clinical translation (Roest et al., 2021).

### 6.1 Normalization challenges in embryo-derived miRNA studies

Normalization of miRNA expression in embryo culture samples remains a key limitation when comparing results across studies. Variations in RNA extraction yield, reverse transcription efficiency, and amplification bias can introduce significant error, especially given the extremely low RNA content of embryo-derived media. Some researchers use exogenous spike-ins, such as cel-miR-39, to adjust for technical differences, while others rely on endogenous reference miRNAs, including miR-16 or miR-191, that appear relatively stable in preimplantation settings. For example, Hawke et al. (2023) evaluated miR-16, miR-191, and miR-106 as stable references for preimplantation mouse embryos (Hawke et al., 2023). In addition, several studies have applied global mean or multi-gene normalization strategies supported by algorithms like NormFinder and geNorm to improve reproducibility. Achieving consensus on standardized normalization approaches will be essential for future clinical translation of embryo-secreted miRNA analyses.

## 7 Future directions

Future research should prioritize the development of standardized protocols for sample collection, RNA isolation, normalization strategies, and data interpretation to improve reproducibility across embryo-derived microRNA studies. The lack of harmonized methodologies remains a major barrier to cross-study comparison and clinical translation, underscoring the need for consensus-driven technical guidelines (Moldovan et al., 2014; Roest et al., 2021).

Prospective, multicenter clinical studies with adequately powered cohorts are essential to validate candidate miRNA panels and to establish clinically actionable thresholds for embryo selection (Rosenbluth et al., 2014; Kamijo et al., 2022). Such validation frameworks will be critical for distinguishing biologically meaningful signals from stress-related or culture-induced miRNA expression.

Technological advances, particularly digital PCR and microfluidic-based platforms, offer promising opportunities for rapid and sensitive detection of low-abundance embryo-secreted miRNAs and may enable same-cycle embryo assessment in IVF laboratories (Whale et al., 2012; Caponnetto et al., 2025). In parallel, integrating miRNA profiling with complementary modalities such as time-lapse imaging, metabolomics, and artificial intelligence–based modeling may enhance predictive accuracy beyond single-parameter approaches (Siristatidis et al., 2021; Gao et al., 2025).

Finally, the translation of miRNA-based embryo assessment tools into routine IVF practice should be guided by robust regulatory frameworks and ethical oversight to ensure embryo safety, data integrity, and equitable clinical implementation (Voros et al., 2025b).

## 8 Conclusion

Accumulating evidence indicates that embryos actively secrete microRNAs into their surrounding environment, particularly into spent culture medium and blastocoel fluid, and that distinct miRNA signatures are associated with chromosomal status and implantation potential. These findings highlight embryo-derived miRNAs as promising non-invasive biomarkers that may complement existing embryo assessment strategies. However, current evidence remains largely observational, and technical challenges, including low RNA yield, contamination risk, and lack of standardized protocols, limit immediate clinical implementation. Continued methodological refinement and large-scale validation will be essential to define the clinical utility of miRNA-based embryo evaluation in IVF.
